# RTMS AND H7-COIL IN THE TREATMENT OF TINNITUS; MODIFIED PROTOCOL PRELIMINARY RESULTS

**DOI:** 10.1192/j.eurpsy.2023.2171

**Published:** 2023-07-19

**Authors:** N. Geres, M. Celic Ruzic, V. Grosic, I. Filipcic

**Affiliations:** University psychiatric clinic Sveti Ivan Zagreb, Zagreb, Croatia

## Abstract

**Introduction:**

A relatively large number of studies of the efficacy of rTMS on tinnitus have been conducted. LF stimulation of the auditory cortex in combination with HF stimulation of the DLPFC, has shown efficacy in reduction of symptoms (especially loudness and anxiety).

**Objectives:**

To launch a protocol to examine the efficacy of Cool-B65 coil LF rTMS combined with H7-deep coil HF rTMS, in the treatment of idiopathic, chronic, normo-acoustic tinnitus disorder, informed by the assessment of efficacy of rTMS on idiopathic, chronic tinnitus disorder. Preliminary results will be presented.

**Methods:**

We first conducted a meta-analysis of randomized sham-controlled, double-blind trials to access the efficacy of rTMS on idiopathic, chronic tinnitus disorder. We are now planning an industry-independent, single-center, prospective, randomized sham-controlled, two-arms, double-blind superiority clinical trial with concealed allocation and masked independent outcome assessment.

Population of this study will include Outpatients diagnosed for ≥ 12 months and ≤ 5 years with persistent, subjective, normo-acoustic tinnitus disorder of at least moderate severity defined by the THI score ≥ 38, both unilateral and bilateral, both genders, and with no hearing loss, age 18-65 years, with the tinnitus treatment unchanged for at least one months. Exclusion criteria will be: organically caused tinnitus and organic brain lesion, objective tinnitus, severe hearing loss or Menier’s disease, middle ear disease, diagnosed mental disorder, suicidality, alcohol or drugs addictions, clinically relevant neurological disorder and standard rTMS exclusion criteria. Population will include 52 in HR rTMS H7-coil arm, and 52 in sham control arm. Adjusted median of differences in instruments measuring tinnitus, annoyance, distress, anxiety, and depression will be used as an outcome.

**Results:**

The Cool-B65 coil LF rTMS (1Hz) placed over the auditory cortex combined with H7-deep coil HF rTMS (10Hz) placed over mPFC, applied for 15 days, is expected to have superior efficacy on tinnitus symptoms measured by the overall THI score, then the SHAM passive coil.

**Conclusions:**

We are conducting a theoretically founded study, with an empirically founded patient-oriented approach. We are expecting that H7-coil in stimulating auditory cortex and mPFC may be a possible alternative target for HF rTMS of the DLPFC.

**Disclosure of Interest:**

None Declared

